# Communication strategies to promote vaccination behaviours in sub-Saharan Africa

**DOI:** 10.1186/s44263-023-00004-7

**Published:** 2023-07-31

**Authors:** Rebecca L. West, Nisa Hurst, Sunny Sharma, Beverley Henry, Shannon Vitale-Rogers, William Mutahi, Frances Salt, Cassie Gardner, Gareth Turley, Nnamdi Ezeanochie, Jason Betik, Sofia Panagiotakopoulou, Janice April, Samantha R. Paige

**Affiliations:** 1 Ipsos, London, UK; 2 Boston University School of Public Health, Boston, MA USA; 3 Ipsos, Nairobi, Kenya; 4 Johnson & Johnson, Health and Wellness Solutions Inc., New Brunswick, NJ USA; 5 Johnson & Johnson, Jacksonville, FL USA; 6 Johnson & Johnson, New Brunswick, NJ USA; 7 Johnson & Johnson, Johannesburg, South Africa

## Abstract

Effective public health communication is needed to increase COVID-19 vaccination uptake in sub-Saharan Africa. We present six best practices: (1) use of collective action/altruism, (2) use of relatable language, (3) tailoring messages, (4) use of gain-framed messages, (5) use of trusted messengers and (6) use of research to test messages.

## Background

COVID-19 vaccine coverage rates lag starkly (https://coronavirus.jhu.edu/vaccines/international) in sub-Saharan Africa compared to the rest of the world, and vaccination levels are well below 60%, the estimated minimum required for herd immunity [1]. Low vaccine uptake in sub-Saharan Africa is due in part to vaccine hesitancy: a recent review found that in one-third of studies, there was lower than 50% acceptance of vaccines among participants in Africa [2]. Vaccine hesitancy is a complex and dynamic social process driven by myriad historical, structural, systemic, political and individual factors [3] and is considered one of the leading threats to global health. Vaccine hesitancy is widely understood to be influenced by factors such as complacency, convenience, and confidence (known as the 3 Cs), and communication is an important tool to address it [3].

One of the three pillars of the regional COVID-19 strategy developed by intergovernmental organisations on the African continent (e.g. the African Union Commission and Africa Centres for Disease Control and Prevention) is to remove barriers to widespread delivery and uptake of the vaccine [1]. Effective public health messaging is a key strategy to ensure uptake when supply is available. However, the time and resources required to develop and test messages are limited and may not be a priority for public health implementers and policymakers. Given the history of slow and inequitable access to medicines during global public health emergencies (e.g. antiretroviral therapy for HIV, H1N1 vaccines [1]) and highly documented levels of vaccine hesitancy and mistrust, it is imperative to bring increased attention and resources to addressing COVID-19 vaccine hesitancy in sub-Saharan Africa. In this comment, we present a list of best practices for COVID-19 vaccination campaign messaging in sub-Saharan Africa (Table 1), based on a review of the literature (specific to sub-Saharan Africa wherever possible) and the authors’ programmatic experience working in global public health. While we focus on sub-Saharan Africa in this comment, these practices may also be applied in other settings.

**Table 1:** Best practices for communication to increase COVID-19 vaccine uptake in sub-Saharan Africa

**Best practice**	**Example message**	Keywords/phrases
1. Use of messages that appeal to collective action and altruism	*The simple act of taking the vaccine protects your family, friends and community at large—especially those who are weak with vulnerable immune systems. Play your part and protect the people you love.*	“Protect” “Play your part”
2. Use of language that is relatable	*All COVID-19 vaccines work with the body’s natural defences to safely develop immunity to disease. That means if you get exposed to the virus after being vaccinated, your body is ready to fight the virus and prevent you from getting sick.*	“Body’s natural defences” “Fight the virus” “Prevent you from getting sick”
3. Tailoring messages to address what drives people to vaccinate	*There are two reasons to get vaccinated: to protect ourselves and protect those around us. Not everyone can be vaccinated including babies or those who have illnesses… they depend on others to be vaccinated to ensure that they are also protected.*	“Protect ourselves and those around us” “Depend on others”
4. Use of gain-framed messages that show the benefits of vaccinating rather than loss-framed messages	*You deserve to chase your dreams, fall in love, start a family and see the world. You deserve to taste success and reap the fruits of your hard work. COVID-19 can stop your dreams from becoming a reality. Get vaccinated and protect your future.*	“You deserve to chase your dreams” “You deserve success” “Protect your future”
5. Use of trusted messengers	–	–
6. Use of research to test messages before they are deployed	–	–

### Use of messages that appeal to collective action and altruism

Prosocial message framing such as evoking altruism has been shown to elicit positive emotions, which may counteract some of the negative emotions associated with vaccination [4]. Depicting vaccination as an individual’s role in upholding collectivist values can also be motivating in collectivist cultures that value social rules promoting selflessness and working as a group. Where COVID-19 morbidity and mortality more heavily impacted the elderly, young people may not relate to the dangers of COVID-19 themselves but understand how it impacts the vulnerable around them.

### Use of language that is relatable

Those who develop messages should consider their target audiences’ literacy levels and the difficulties they may face accessing information. The messages should employ language that is easily understood rather than using overly technical terms, which may cause confusion [4]. Engaging with key stakeholders and community members in message design can ensure that information is relatable [4] and can identify culturally appropriate ways to explain scientific concepts.

### Tailoring messages to address what drives people to vaccinate

Public information messages without a call to action or emotional appeal have been proven insufficient to motivate someone to vaccinate [5]. Instead, message tailoring is the optimal approach to delivering persuasive messages for health behaviour change in the context of COVID-19 [4]. Tailoring messages requires identifying the unique individual barriers and behavioural factors that impact a desired behaviour (such as vaccinating) and then focusing communication to address these barriers by giving this person the specific information they need [6]. Each individual’s life experiences, culture, personal background, political leanings, and religion all influence their decisions to vaccinate [5]. Tailored messages should appeal to people on an individual level, connecting the decision to vaccinate with what matters most to them. For example, tailored messages could focus on the benefits of vaccination for individuals, their loved ones and communities.

### Use of gain-framed messages that show the benefits of vaccinating rather than loss-framed messages

Health messages may be framed in terms of the benefits of a health behaviour (gain-framed) or the detrimental consequences of an unhealthy one (loss-framed) [7]. In the context of COVID-19, gain-framed messages may be more impactful than loss-framed messages. Widespread anxiety, loss and psychological fatigue caused by the COVID-19 pandemic should be treated with care, and communications should attend to those emotions rather than heighten them [8]. Behaviour change researchers recommend framing messages in a way that emphasises self-determined motives and intrinsic goals in order to increase the likelihood that behaviours are internalised [9]. For example, getting vaccinated can be framed as a way of taking agency and allowing one to plan for the future, which may be resonant after a long period of feeling powerless throughout the pandemic due to lockdown measures that may have taken away economic and social opportunities.

### Choice of trusted messengers

There is a role for both experts and non-experts to deliver COVID-19 vaccine messages. Expert messengers should be used to deliver medical and technical information, such as explaining how vaccines work and why they are safe. Lack of trust in government and its negative impact on risk communication and community engagement has been widely documented across sub-Saharan Africa during the pandemic [10]. The most trusted experts are likely to be those with healthcare backgrounds rather than government officials.

In contrast, non-experts are best positioned to deliver messages about community support and the impact of vaccination on one’s personal life. The ideal non-expert messengers are those who serve as trusted sources of information and advisors in decision-making. Research in sub-Saharan Africa suggests that non-governmental and civil society organisations and influential religious and cultural leaders play a crucial role in motivating communities towards trust in and acceptance of vaccination [5]. Their messages can reinforce how vaccinating does not conflict with local norms and customs. It is important to consider that non-expert messengers will require their own communication supports, to have informed conversations and provide accurate information about vaccination.

Expert and non-expert messengers do not have to work in silos. There is a need to deliver messages from healthcare experts to people outside of health facilities. Campaigns should consider pairing healthcare providers and experts with non-experts such as community or religious leaders to boost message campaigns’ visibility and appeal.

### Use of research to test messages before they are deployed

Researchers should think creatively about how to evaluate which messages may be preferred in different settings. Cross-sectional surveys can be used to measure both openness to vaccinate (mindset) and future intention to vaccinate (action) following exposure to different messages. Including a baseline measurement of openness and intention to vaccinate can also help to understand how messages may move the needle for each person. Qualitative methods can be leveraged to pilot test tools, explore how messages resonate with different target audiences or generate recommendations for adaptation and refinement.

## Conclusions

Getting health messages right during the COVID-19 pandemic has been a challenge around the world as our understanding of the virus, preventive measures, and treatments have changed over time, and misinformation and politicisation of prevention measures have been rampant. Most of the research into communication strategies to improve COVID-19 vaccine acceptance has been conducted in the Global North. The lag in vaccination uptake and documented widespread hesitancy in sub-Saharan Africa indicates an urgent need for impactful public health messaging to reach protective levels in sub-Saharan Africa [1] and to promote equitable access of vaccines globally. Designing impactful COVID vaccination messages and campaigns requires a two-pronged strategy: for public health practitioners and policymakers alike to draw on lessons learned from behavioural science and health communications and for researchers to employ quantitative and qualitative tools to further refine our understanding of what works best to address COVID-19 vaccine hesitancy in sub-Saharan Africa and similar settings.

## Data Availability

Not applicable.
